# Fatigue and associated factors among patients with systemic lupus erythematosus in China: a cross-sectional study

**DOI:** 10.3389/fmed.2025.1688619

**Published:** 2025-12-05

**Authors:** Hong Zhang, Qingfang Wu, Dehui Cai, Buyue Shi, Yali Zhu, Qiong Huang

**Affiliations:** 1 Nursing Department, Minda Hospital of Hubei Minzu University, Enshi, Hubei, China; 2 Hubei Provincial Key Laboratory of Occurrence and Intervention of Rheumatic Diseases (Hubei Minzu University), Enshi, Hubei, China; 3 Physical Examination Center, Minda Hospital of Hubei Minzu University, Enshi, Hubei, China; 4 Department of Rheumatology and Immunology, Minda Hospital of Hubei Minzu University, Enshi, Hubei, China; 5 Department of Rheumatology and Immunology, Enshi Huiyi Hospital of Rheumatic Diseases, Enshi, Hubei, China

**Keywords:** systemic lupus erythematosus, fatigue, social support, pain, depression, sleep quality

## Abstract

**Purpose:**

This study aimed to evaluate fatigue in systemic lupus erythematosus (SLE) patients and systematically analyze the main factors associated with fatigue.

**Methods:**

We recruited potential participants from the Department of Rheumatology and Immunology of two tertiary hospitals in China between August 2021 and January 2022. We used questionnaires to collect research data, including sociodemographic data, disease-related data, fatigue, anxiety and depression, illness perception, social support, sleep quality, physical activity, and disease activity. The independent sample t-test, one-way analysis of variance (ANOVA), non-parametric test, Pearson’s/Spearman’s correlation analysis, and multiple linear regression analysis were used in this study.

**Results:**

A total of 201 patients with SLE were included in this study. The prevalence of fatigue in SLE patients was 58.7%, with a mean fatigue score of 4.36 ± 1.18. The multiple linear regression analysis revealed that higher depression (*β* = 0.238, *p* < 0.001), higher illness perception (*β* = 0.143, *p* = 0.005), more pain (*β* = 0.243, *p* < 0.001), and worse sleep quality (*β* = 0.231, *p* < 0.001) were associated with worse fatigue, but higher social support (*β* = −0.291, *p <* 0.001) and physical activity (*β* = −0.096, *p* = 0.024) were associated with lower fatigue. Monthly household income per capita and educational level were also associated with fatigue (all *p* < 0.05).

**Conclusion:**

The prevalence of fatigue in SLE was 58.7%. Fatigue was associated with monthly household income, educational level, depression, illness perception, pain, social support, sleep quality, and physical activity. No significant association was observed between anxiety, disease activity, age, work status, and fatigue. Future fatigue management for SLE patients should prioritize modifiable non-disease-activity-related factors.

## 1 Introduction

Systemic lupus erythematosus (SLE) is a chronic systemic autoimmune disease characterized by periods of remission and recurrence (1). The global prevalence of SLE is approximately 0–241/100,000, and the prevalence of SLE in China is approximately 30 ~ 70/100,000 (2, 3). SLE is more prevalent among women than men, with a male-to-female ratio of approximately 1:10–12 (1). SLE leads to a wide range of clinical manifestations and symptoms, and fatigue is one of the most common symptoms (4). A systematic review, including 16 studies from 8 countries, revealed that the prevalence of fatigue in SLE patients was 65.8% (5).

Fatigue has been defined as a subjective, unpleasant sensation of exhaustion with both physical and mental components, which interferes with work ability and impairs quality of life (4, 6). Fatigue may negatively affect several key aspects of overall quality of life, including physical health, mental health, and social function (6). Thus, healthcare professionals should identify factors associated with fatigue and develop targeted interventions for reducing fatigue in SLE patients.

Davies et al. (7) summarized that biological, physiological, and psychosocial mechanisms contribute to fatigue. Previous studies (8–15) have explored a variety of factors related to fatigue, such as pain, sleep quality, anxiety, depression, social support, and illness perception. However, these studies found inconsistent results. Pinto et al. (13) believed that both disease course and disease activity had an impact on fatigue; however, other studies (16, 17) found no significant difference in the prevalence of fatigue among SLE patients with different disease courses and disease activities. The severity of fatigue in SLE patients is correlated with age (18), educational level (14), and socioeconomic status (19). In contrast, Omdal et al. (20) found that fatigue was not correlated with sociodemographic variables in SLE patients.

Some studies (17, 19) have only explored a few factors associated with fatigue. Du et al. (17) conducted a single-center, cross-sectional study and only explored the relationships between disease duration, depression, anxiety, sleep quality, and fatigue. Although Davies et al. (7) proposed a conceptual framework describing the mechanisms and determinants of fatigue, published studies (4, 16, 21) did not build upon this framework when selecting and examining potential influencing factors of fatigue. Additionally, the relationships between sociodemographic factors, disease-related factors, psychological factors, and fatigue in Chinese patients with SLE have not been well studied. Limited studies on fatigue and its associated factors hinder the development of effective and targeted interventions for addressing fatigue in SLE patients. Thus, this study aimed to evaluate fatigue in Chinese SLE patients and systematically analyze the comprehensive factors associated with fatigue.

### 1.1 Conceptual framework

**Figure 1:**
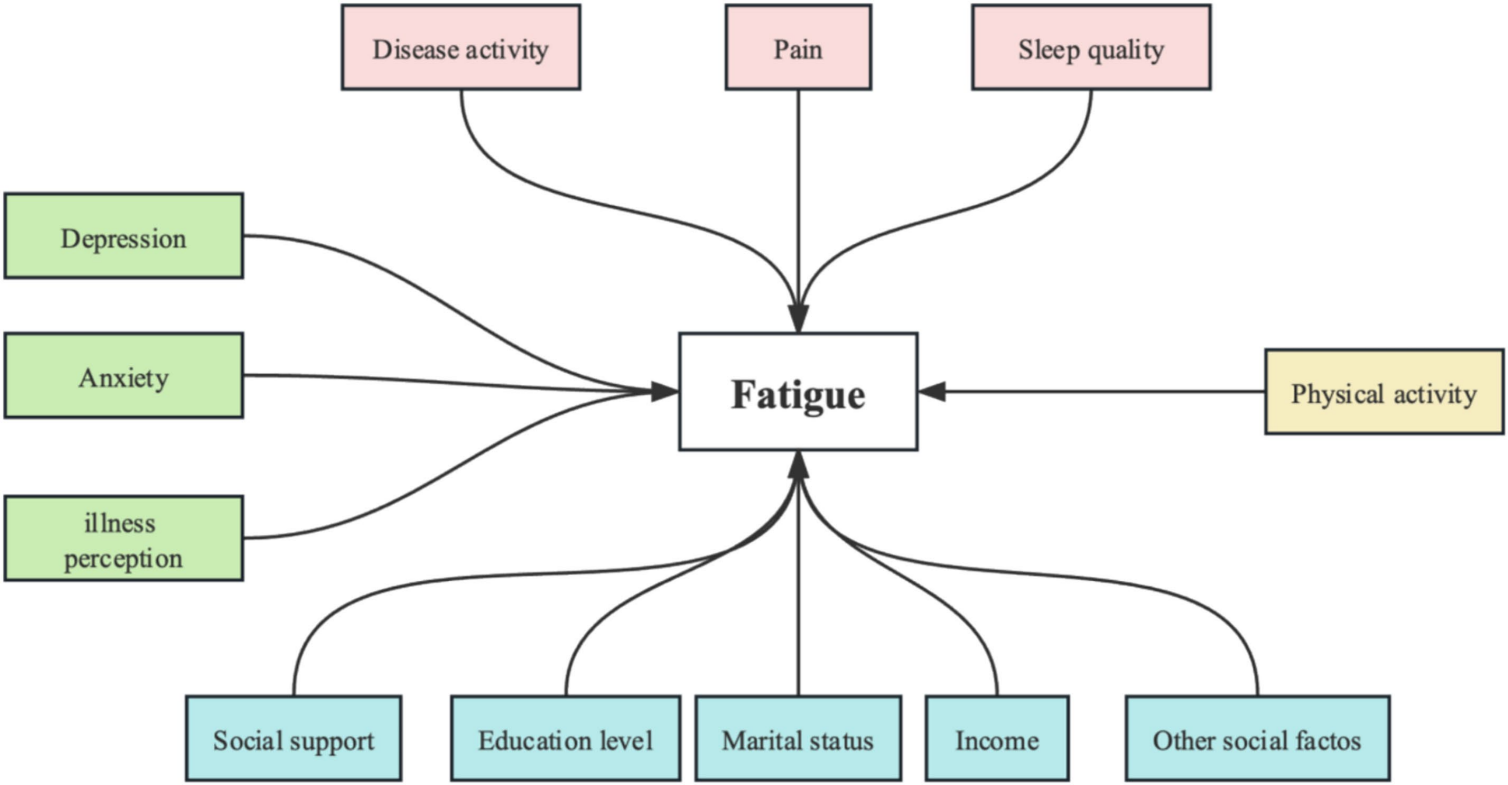
Conceptual framework of this study. Putative determinants of fatigue include biological factors (e.g., disease activity, pain, and sleep quality), psychological factors (e.g., depression, anxiety, and illness perception), behavioral factors (e.g., physical activity), and social factors (e.g., social support, education level, marital status, income, and other social variables). These factors may contribute to developing fatigue and interact with each other.

This study was guided by the mechanistic and conceptual models of fatigue proposed by Davies et al. (7) and aimed to explore the comprehensive factors associated with fatigue. Davies et al. (7) outlined various determinants of fatigue in inflammatory rheumatic diseases and proposed mechanistic and conceptual models of fatigue. Fatigue pathogenesis involves various biological, physiological, and psychosocial mechanisms (7). The psychosocial or behavioral factors involve anxiety, depression, illness perception, pain, and physical activity (7, 13). A previous study (22) also revealed that adverse life events (whether in early life or adulthood), access to psychosocial support, relationship status, income, and educational levels are associated with fatigue in chronic diseases. Biological or physiological factors include inflammation, sleep disturbances, mood disturbances (7), and pain. According to the mechanistic and conceptual model of fatigue, we aimed to identify the determinants of fatigue in SLE patients, including biological or physiological factors (disease activity, pain, and sleep quality), psychological factors (anxiety, depression, and illness perception), social factors (social support, education level, work status, ethnicity, marital status, income, and other social factors), and behavioral factors (physical activity). Figure 1 describes the conceptual framework.

## 2 Methods

### 2.1 Study design and participants

This study aimed to explore fatigue in SLE patients and systematically analyze the comprehensive factors associated with fatigue. This multicenter cross-sectional study was conducted from August 2021 to January 2022.

We trained investigators from each hospital on how to recruit potential participants and collect questionnaires. The investigators used convenience sampling to recruit potential participants in the departments of Rheumatology and Immunology of two hospitals during their routine care. Investigators assessed potential participants, obtained their approval, and distributed questionnaires to them. The inclusion criteria for participants were: (1) meeting the American College of Rheumatology (ACR) classification criteria for SLE, (2) aged ≥ 18 years, (3) able to clearly understand and communicate in Chinese, and (4) willing to participate in this study. The exclusion criteria for participants were: (1) pregnancy; (2) presence of other rheumatic diseases, such as rheumatoid arthritis, Sjögren’s disease, scleroderma, or fibromyalgia; and (3) presence of other severe diseases, such as neurological diseases or psychiatric disorders.

### 2.2 Ethical consideration

This study adhered to the principles outlined in the Declaration of Helsinki. We obtained ethical approval from the Medical Ethics Committee of Hubei Minzu University (ID: 202163). All participants were informed of the purpose of this study, and they had the right to withdraw from the study voluntarily at any time. Written informed consent was obtained from all participants at the start of the study.

### 2.3 Instrument

We used questionnaires to collect patients’ demographic and disease-related information, disease activity, fatigue, anxiety, depression, illness perception, pain, social support, sleep quality, and physical activity.

#### 2.3.1 Demographic and disease-related data

Demographic data include sex, age, education level, work status, ethnicity, marital status, monthly household income per capita, and other relevant factors. Disease-related data include medication use and comorbidities. Pain was measured using a 10-cm horizontal visual analog scale, with higher scores indicating a higher level of pain. Disease Activity was measured by using the Systemic Lupus Erythematosus Disease Activity Index 2000 (SLEDAI-2000) scale (1). This scale includes 24 items with a total score ranging from 0 to 105. According to the SLEDAI-2000 score criteria, disease activity can be classified as remission (SLEDAI-2000 = 0), mild activity (1SLEDAI-2000 ≤ 6), moderate activity (7 ≤ SLEDAI-2000 ≤ 12), and severe activity (SLEDAI-2000 > 12) (23).

#### 2.3.2 Fatigue

Fatigue was assessed using the Fatigue Severity Scale (FSS), developed by Krupp et al. (24). FSS includes nine items designed to assess the impact of fatigue on specific types of functioning over the previous 2 weeks. Each item is rated on a 7-point Likert scale, ranging from 1 (strongly disagree) to 7 (strongly agree). The overall score is calculated as the average of the 9 items, where a mean score of 4 or higher indicates the presence of fatigue (25). Higher scores indicate more severe fatigue. The Chinese version of FSS is a valid and reliable tool for evaluating fatigue. Wu et al. (26) reported that exploratory factor analysis explained 61.89% of the total item variance in the Chinese version of the FSS, with a Cronbach’s *α* of 0.9287. In our pilot study, the Cronbach’s α of the scale was 0.897.

#### 2.3.3 Illness perception

The illness perception of SLE patients was assessed using the Chinese version of the Brief Illness Perception Questionnaire (BIPQ) (27). The BIPQ is a 9-item scale designed to assess patients’ cognitive and emotional representations of their illness (28), with all items rated on a 0-to-10 response scale. Five items assess cognitive representations of illness, two items assess emotional representations, and one item assesses illness comprehensibility. The ninth item asks patients to list the three most important causes of their illness. The total BIPQ score ranges from 0 to 80, with higher scores indicating a stronger pronounced perception of illness across each dimension. The Cronbach’s *α* coefficient of the Chinese version of the BIPQ was 0.77 (27), and in our pilot study, the Cronbach’s α was 0.818.

#### 2.3.4 Sleep quality

Participants’ sleep quality was assessed using the Chinese version of the Pittsburgh Sleep Quality Index (PSQI) (29). The PSQI comprises seven dimensions: subjective sleep quality, sleep latency, sleep duration, habitual sleep efficiency, sleep disorders, use of hypnotics, and daytime dysfunction (30). The PSQI score ranges from 0 to 21, with higher scores indicating poorer sleep quality (30). The Cronbach’s *α* of the Chinese version of PSQI was 0.8424, and the test–retest reliability was 0.8092 (29). Clinically, a PSQI score ≤ 7 is considered normal, while a score > 7 indicates a sleep disorder (29). The Cronbach’s *α* coefficient of the PSQI in our pilot study was 0.801.

#### 2.3.5 Anxiety and depression

Anxiety and depression were measured using the Hospital Anxiety and Depression Scale (HADS) (31). The HADS is a reliable tool for screening for depression and anxiety in a medical outpatient setting. The scale consists of 14 items, divided into 2 subscales: an anxiety subscale and a depression subscale. The subscale score ranges from 0 to 21, and a higher score indicates worse anxiety and depression (31). The anxiety subscale and depression subscale scores >7 are considered as depression symptoms or anxiety symptoms in the participants (32). The Cronbach’s *α* of the Chinese version of the HADS and its subscales were 0.879 and 0.879, respectively (32). In this study, the Cronbach’s α of anxiety and depression subscales were 0.779 and 0.797, respectively.

#### 2.3.6 Physical activity

The International Physical Activity Questionnaire, Short Form, Chinese version (IPAQ-S-C) was used to measure participants’ physical activity (33). The IPAQ-S records self-reported physical activity in the last 7 days. Responses were converted into Metabolic Equivalent Task minutes per week (METmin/wk). Total minutes spent on vigorous activity, moderate-intensity activity, and walking over the last 7 days were calculated into MET scores for each activity level (34). This questionnaire has been used to measure the physical activity level of SLE patients and has demonstrated good reliability and validity (35). The intraclass correlation coefficient (ICC) of the Chinese version of the IPAQ-S-C was 0.79 (36).

#### 2.3.7 Social support

The Perceived Social Support Scale (PSSS) was used to assess participants’ social support (37). The 12-item PSSS is used to measure an individual’s perceived support from family, friends, and significant others. The scores of the subscales and total scale range from 1 to 7, with a higher score indicating higher perceived social support. The Cronbach’s *α* for the Chinese version of PSSS was 0.840, and the split-half reliability coefficients ranged from 0.741 to 0.791 (38). In our pilot study, the Cronbach’s α of PSSS was 0.851.

### 2.4 Data collection

The SLEDAI questionnaires were obtained from both investigators and participants’ medical records. Participants independently completed the self-reported questionnaires. If they had questions regarding the questionnaires, the investigators provided explanations and assistance to facilitate completion. The investigators reviewed the questionnaires for accuracy and completeness after participants had completed them.

### 2.5 Statistical analysis

Data analysis was conducted using IBM SPSS Statistics version 26.0 (IBM Corp., Armonk, NY, USA). The independent sample t-test, one-way ANOVA, non-parametric test, and Pearson’s or Spearman’s correlation analysis were used to explore the relationships between fatigue and other variables. The least significant difference (LSD) method was used for post-hoc comparisons of variables that exhibited significant differences in the one-way ANOVA. Finally, multiple linear regression analysis was used to explore the factors associated with fatigue in SLE patients.

## 3 Results

### 3.1 Participants’ characteristics

We distributed 235 questionnaires and collected 201 questionnaires. The mean age of the participants was 37.23 years (SD = 11.17), ranging from 18 to 77 years. The majority of participants were female (96.5%, *N* = 194), of Han ethnicity (*N* = 107, 53.2%), and were either married or cohabiting (*N* = 150, 74.6%). They had a senior high school educational level or above (*N* = 110, 54.7%). More than half (*N* = 107, 53.2%) of the participants had a per capita monthly household income of ≤5,000 RMB, and 35.8% (*N* = 72) had a per capita monthly household income of ≤2000 RMB. The demographic and disease-related characteristics of participants are shown in Table 1.

**Table 1:** General characteristics and univariate analysis of variables associated with fatigue of the participants (*n* = 201).

Variable	Category	*N*	FSS score (mean ± SD)	*F/t*	*p*
Age (years, mean ± SD)	37.23 ± 11.17	201	4.36 ± 1.18		
	18 ~ 34	99	4.35 ± 1.18	4.043^a^	**0.008**
	35 ~ 44	56	4.18 ± 1.03		
	45 ~ 54	30	4.96 ± 1.19		
	55~	16	3.89 ± 1.33		
Sex	Female	194	4.37 ± 1.19	0.853^b^	0.395
	Male	7	3.98 ± 1.08		
Educational level	Primary school or below	27	4.93 ± 1.31	3.312^a^	**0.021**
	Junior high school	64	4.44 ± 1.15		
	Senior high school/secondary vocational school	56	4.22 ± 1.10		
	College or above	54	4.12 ± 1.16		
Monthly household income per capita (RMB, yuan)	≤2000	72	4.78 ± 1.18	8.843^a^	**<0.001**
	2001 ~ 5,000	35	4.56 ± 1.34		
	5,001 ~ 8,000	25	4.33 ± 0.94		
	>8,000	69	3.83 ± 0.98		
Work status	Work	72	4.11 ± 1.05	2.962 ^a^	**0.033**
	Self-employment/farmers	31	4.45 ± 1.19		
	Retirement	15	3.97 ± 1.19		
	Unemployed	83	4.61 ± 1.25		
Ethnicity	Ethnic minority	94	4.43 ± 1.24	0.743^b^	0.458
	Han	107	4.30 ± 1.14		
Marital status	Married/cohabitation	150	4.35 ± 1.22	−0.067^b^	0.867
	Others (separation/divorced/widowed)	51	4.38 ± 1.09		
Disease activity	Remission	13	3.93 ± 1.02	4.453^a^	**0.005**
	Mild activity	128	4.23 ± 1.13		
	Moderate activity	47	4.57 ± 1.26		
	Serious activity	13	5.28 ± 1.09		

SD, standard deviation.

^a^One-way ANOVA.

^b^Independent sample t-test.

Bold indicates *p* < 0.05.

### 3.2 Fatigue and related variables

The mean fatigue score among SLE patients was 4.36 (SD = 1.18), and scores ranged from 1.67 to 7.00. The prevalence of fatigue within this population was 58.7%. The mean scores of anxiety, depression, illness perception, social support, and sleep quality were 6.28, 9.68, 46.39, 50.11, and 7.73, respectively. Sixty-eight (33.8%) patients reported anxiety symptoms, 143 (71.1%) reported depressive symptoms, and 100 (49.8%) exhibited sleep disturbances. The median SLEDAI score was 4, and 93.5% of patients reported disease activity. The median score for physical activity was 1,386. The scores for fatigue and other variables are described in Tables 1, 2.

**Table 2:** Correlations between continuous variables and fatigue.

Variables	Mean±SD/ Median (P_25_, P_75_)	Range	*r*	*p*
Anxiety	6.28 ± 2.56	1 ~ 14	0.177	**0.012**
Depression	9.68 ± 4.10	1 ~ 20	0.560	**<0.001**
Illness perception	46.39 ± 13.06	6 ~ 73	0.559	**<0.001**
Social support	50.11 ± 16.53	12 ~ 82	−0.489	**<0.001**
Sleep quality	7.73 ± 3.37	0 ~ 17	0.470	**<0.001**
Disease activity	4(2, 8)	0 ~ 36	0.288	**<0.001**
Pain	4.08 ± 2.06	0 ~ 9	0.539	**<0.001**
Physical activity	1,386(693, 2,772)	330 ~ 13,545	−0.302	**<0.001**

SD, standard deviation.

Bold indicates *p* < 0.05.

### 3.3 Factors associated with fatigue

In univariate analysis, the mean score of FSS exhibited significant differences across various demographic factors, including age (*F* = 4.043, *p* = 0.008), educational level (*F* = 3.312, *p* = 0.021), monthly household income per capita (*F* = 8.843, *p* < 0.001), and work status (*F* = 2.962, *p* = 0.033). Pearson’s and Spearman’s rank analyses found that FSS score was positively associated with anxiety (*r* = 0.177, *p* = 0.012), depression (*r* = 0.560, *p* < 0.001), illness perception (*r* = 0.559, *p* < 0.001), sleep quality (*r* = 0.470, *p* < 0.001), pain (*r* = 0.539, *p* < 0.001), and disease activity (*r* = 0.288, *p* < 0.001), but FSS score was negatively associated with social support (*r* = −0.489, *p* < 0.001), and physical activity (*r* = −0.302, *p* < 0.001).

The results of multiple linear regression analyses using FSS total scores as dependent variables are shown in Table 3. The results showed that higher depression (*β* = 0.238, *p* < 0.001), higher illness perception (*β* = 0.143, *p* = 0.005), more pain (*β* = 0.243, *p* < 0.001), and worse sleep quality (*β* = 0.231, *p* < 0.001) were associated with higher fatigue. We also found that higher social support (*β* = −0.291, *p* < 0.001) and physical activity (*β* = −0.096, *p* = 0.024) were associated with lower fatigue. Educational level and monthly household income per capita were also associated with fatigue. Participants with an educational level of senior high school or higher reported lower fatigue compared with those with a primary school educational level (all *p* < 0.05). Patients with a higher monthly household income per capita (approximately 8,000 yuan) reported lower fatigue compared with those with the lowest monthly household income per capita (≤2000 yuan). The adjusted *R*^2^ of this model was 0.69, indicating that these variables could explain 69.0% of the variance in fatigue.

**Table 3:** Multiple linear regression analysis of variables associated with fatigue.

Variables	*B*	SE	β	*t*	*p*	95%CI	
Anxiety	−0.148	0.182	−0.036	−0.811	0.418	−0.508	0.212
Depression	0.617	0.131	0.238	4.711	**<0.001**	0.359	0.876
Illness perception	0.117	0.041	0.143	2.830	**0.005**	0.035	0.198
Pain	1.258	0.238	0.243	5.293	**<0.001**	0.789	1.727
Social support	−0.187	0.029	−0.291	−6.382	**<0.001**	−0.245	−0.129
Sleep quality	0.730	0.145	0.231	5.030	**<0.001**	0.443	1.016
Physical activity	0.000	0.000	−0.096	−2.275	**0.024**	−0.001	0.000
Disease activity	0.133	0.090	0.063	1.481	0.140	−0.044	0.310
Age (year)							
18 ~ 34	Reference						
35 ~ 44	−0.876	1.077	−0.037	−0.813	0.417	−3.002	1.250
45 ~ 54	1.246	1.424	0.042	0.875	0.383	−1.564	4.055
55~	−2.965	2.373	−0.076	−1.250	0.213	−7.646	1.717
Educational level							
Primary school or below	Reference						
Junior high school	−2.579	1.487	−0.113	−1.734	0.085	−5.514	0.356
Senior high school/secondary vocational school	−2.961	1.475	−0.125	−2.008	**0.046**	−5.871	−0.051
College or above	−3.084	1.631	−0.129	−1.890	0.060	−6.303	0.136
Work status							
Employment	Reference						
Self-employment/farmers	0.632	1.509	0.021	0.419	0.676	−2.346	3.610
Retirement	3.236	2.585	0.082	1.252	0.212	−1.864	8.337
Unemployed	2.244	1.140	0.104	1.969	0.051	−0.005	4.494
Monthly household income per capita (RMB, yuan)							
≤2000	Reference						
2001 ~ 5,000	−0.374	1.324	−0.013	−0.283	0.778	−2.986	2.238
5,001 ~ 8,000	1.540	1.460	0.048	1.055	0.293	−1.341	4.421
8,001~	−2.392	1.109	−0.107	−2.156	**0.032**	−4.581	−0.203
Constant	29.887	3.553		8.413		22.877	36.897
*R*^2^ = 0.721, Adjusted *R*^2^ = 0.690							

CI, confidence interval.

Bold indicates *p* < 0.05.

## 4 Discussion

This study found that 58.7% of patients with SLE reported fatigue, which was similar to previous studies (5, 19). Liu et al. (5) conducted a meta-analysis and reported a 65.8% fatigue rate in SLE patients. Although the prevalence of fatigue in the current study is similar to that reported previously, different FFS cutoff values were applied. Some studies included in Liu et al.’s meta-analysis defined the presence of fatigue using an FSS score of ≥3 (5). SLE patients have a high prevalence of fatigue, which may seriously affect their physical and mental health and social functioning (4). The current study employed the conceptual models of fatigue proposed by Davies et al. (7) alongside relevant literature to identify the potential factors associated with fatigue. We found that monthly household income per capita, educational level, pain, depression, sleep quality, illness perception, physical activity, and social support were significantly associated with fatigue. The adjusted R^2^ was 0.690, indicating that the model (comprising biological or physiological factors, social factors, and behavioral factors) explains a substantial majority of the variance in fatigue. We suggested that future fatigue management for SLE patients should prioritize modifiable non-disease-activity-related factors, such as pain, depression, sleep quality, illness perception, physical activity, and social support.

The current study found that patients with higher monthly household income per capita (approximately 8,000 yuan) and a higher educational level reported lower fatigue. Feng et al. (19) demonstrated that patients with lower income levels experienced higher fatigue compared to those with higher family incomes. Similarly, Moldovan et al. (39) revealed that the family income and educational level of SLE patients were negatively associated with fatigue. SLE is a chronic rheumatology disease requiring long-term treatment, which may put a disease burden on patients and make them feel fatigued (40). Patients with a higher level of education can better understand disease management, access healthcare resources, and adopt healthier behaviors (41). However, patients with lower income levels are often linked to delayed diagnosis, restricted access to specialized care, and limited utilization of treatment options (41). These may be possible reasons why patients with higher educational attainment and income report lower levels of fatigue. Thus, healthcare professionals should pay closer attention to individuals with low educational attainment and income.

In the current study, higher pain was associated with a higher level of fatigue. Pinto et al. (13) and Li et al. (42) also concluded that pain was one of the independent factors influencing fatigue in SLE patients. Patients with higher pain levels suffered depression, more fatigue, and impaired quality of life. In addition, pain contributes to reduced physical activity and muscle dysfunction, which may increase perceived fatigue or exacerbate pain (43, 44).

Our study showed that depression was positively associated with fatigue. Omdal et al. (45) revealed that depression is an important predictor of fatigue. The prevalence of depression in SLE patients has been reported to be higher than that in the general population (21). Karol et al. (23) found that 41.7% of patients reported moderate to severe depressive symptoms. Da Costa et al. (46) showed that both physical and mental fatigue were related to depression, and depression was a stronger determinant of mental fatigue. The course of SLE is usually unpredictable, with remission and recurrence (1). During the long-term disease course, patients may lack knowledge of disease management and lose confidence in controlling SLE, which may lead to depression and fatigue. Therefore, healthcare professionals should incorporate emotional management into their fatigue interventions.

This study found that poor sleep quality was associated with severe fatigue. A previous study (46) has also indicated that sleep disorders contribute to the onset of fatigue in SLE patients. Studies (46, 47) have highlighted that sleep disorders, arising from both physiological and psychological factors, are associated with increased fatigue levels. When patients experience sleep disorders, their body’s energy is significantly depleted, leading to an increased perception of fatigue (47). Thus, sleep intervention is important for healthcare professionals to manage fatigue among SLE patients.

The current study found that higher physical activity levels were associated with lower levels of fatigue in SLE patients. Previous studies (35, 46) have also shown that reduced physical activity is associated with higher levels of fatigue. Longer durations of moderate or high-intensity physical activity are correlated with reduced fatigue (48, 49). Physical activity had the smallest standardized effect size among all significant factors associated with fatigue. Our results indicated that the impact of physical activity on fatigue was relatively weaker compared with other factors, such as social support and pain. Nevertheless, physical activity remains an important modifiable factor. Thus, healthcare professionals should focus on the most important factors (e.g., pain and social support) associated with fatigue and develop appropriate physical programs for SLE patients.

We found that higher illness perception was associated with severe fatigue. Nowicka et al. (15) demonstrated a significant association between illness perception and fatigue. Fatigue may both result from and contribute to a poorly perceived health status (15). Similarly, Lu et al. (21) identified correlations between fatigue and various variables, such as symptom perception, timeline (chronic or cyclical), consequences, and coherence (understanding of the illness), as well as the severity of negative emotions. Furthermore, patients’ perception of chronic disease duration, unpredictable symptoms, and its consequences may lead to the perception of worse physical health and fatigue (50). These findings suggest that promoting positive illness perceptions among patients may be a potential method to alleviate fatigue in SLE patients.

We also found that social support was negatively associated with fatigue. Fatigue is inversely related to perceived levels of social support (12). Enhancing social support represents a potentially modifiable strategy to improve both physical and psychological functioning in patients, which may help reduce fatigue (51). Previous studies (49, 52) revealed that support and comfort from family and the social environment serve as protective factors against fatigue. Thus, social support from family and the social environment is crucial for individuals to reduce their fatigue.

In this study, anxiety, disease activity, age, and work status were found to be significantly associated with fatigue in the univariate analysis. However, these factors were not included in the multiple linear regression model as independent variables. It is common for a variable to be highly significant in univariate analysis yet have no role in multiple regression (53). The possible reason was that the initial univariate analysis ignored the correlations among variables (53). Depression, illness perception, anxiety, physical activity, and other biological and physiological factors are direct or indirect determinants of fatigue (7). According to the Common-Sense Model of Self-Regulation, patients are active agents who dynamically construct their own subjective understanding of an illness, which in turn directly governs their coping strategies and emotional experiences (50). In the current study, anxiety may indirectly affect fatigue through illness perception. Mertz et al. (16) revealed that fatigue in SLE patients was not associated with disease activity, as assessed using SLEDAI. Symptoms such as fatigue, depression, and anxiety are often unresponsive to immunosuppressive therapy (16). Thus, disease activity may not be significantly associated with fatigue.

### 4.1 Limitations

The current study had several limitations. First, this study was conducted at only two hospitals in China. However, the study’s findings may be limited by the inclusion of participants from only two hospitals. Future research could conduct nationwide multicenter studies. Second, a key limitation of this study was that weused self-reported measures to assess fatigue, pain, depression, sleep quality, and other variables. These subjective outcomes may not strongly correspond to specific biological markers. Further studies should incorporate the biological markers associated with fatigue in SLE patients. Finally, the cross-sectional design of this study does not allow for causal interpretation. Future research should use longitudinal study designs to explore the predictors of fatigue.

## 5 Conclusion

The prevalence of fatigue in SLE patients was 58.7%. Fatigue was associated with multiple factors, including family income, educational level, pain, sleep quality, depression, illness perception, social support, and reduced physical activity. However, no significant associations were observed with anxiety, disease activity, age, work status, or fatigue. Future fatigue management for SLE patients should prioritize modifiable non-disease-activity-related factors, such as pain and social support.

## Data Availability

The raw data supporting the conclusions of this article will be made available by the authors, without undue reservation.

## References

[1] Li M Zhao Y Zhang Z Huang C Liu Y Gu J . 2020 Chinese guidelines for the diagnosis and treatment of systemic lupus erythematosus. Rheumatol Immunol Res. (2020) 1:5–23. doi: 10.2478/rir-2020-0009, PMID: 36465077 PMC9524765

[2] Rees F Doherty M Grainge MJ Lanyon P Zhang W. The worldwide incidence and prevalence of systemic lupus erythematosus: a systematic review of epidemiological studies. Rheumatology. (2017) 56:1945–61. doi: 10.1093/rheumatology/kex260, PMID: 28968809

[3] Zeng Q Chen R Darmawan J Xiao Z Chen S Wigley R . Rheumatic diseases in China. Arthritis Res Ther. (2008) 10:R17. doi: 10.1186/ar2368, PMID: 18237382 PMC2374446

[4] Kawka L Schlencker A Mertz P Martin T Arnaud L. Fatigue in systemic lupus erythematosus: an update on its impact, determinants and therapeutic management. J Clin Med. (2021) 10:3996. doi: 10.3390/jcm10173996, PMID: 34501444 PMC8432566

[5] Liu J Zhang M Li X. Systematic evaluation of the incidence of fatigue in patients with svstemic lupus erythematosus. Chinese Evidence-Based Nursing. (2021) 7:1884–92. doi: 10.12102/j.issn.2095-8668.2021.14.006

[6] Hardy SE Studenski SA. Qualities of Fatigue and Associated Chronic Conditions Among Older Adults. J Pain Symptom Manag. (2010) 39:1033–42. doi: 10.1016/j.jpainsymman.2009.09.026, PMID: 20538185 PMC2884149

[7] Davies K Dures E Ng W-F. Fatigue in inflammatory rheumatic diseases: current knowledge and areas for future research. Nat Rev Rheumatol. (2021) 17:651–64. doi: 10.1038/s41584-021-00692-1, PMID: 34599320

[8] Waldheim E Ajeganova S Bergman S Frostegård J Welin E. Variation in pain related to systemic lupus erythematosus (SLE): a 7-year follow-up study. Clin Rheumatol. (2018) 37:1825–34. doi: 10.1007/s10067-018-4079-1, PMID: 29654486 PMC6006213

[9] Azizoddin DR Jolly M Arora S Yelin E Katz P. Longitudinal Study of Fatigue, Stress, and Depression: Role of Reduction in Stress Toward Improvement in Fatigue. Arthritis Care Res. (2020) 72:1440–8. doi: 10.1002/acr.24052, PMID: 31421030 PMC7024647

[10] Figueiredo-Braga M Cornaby C Cortez A Bernardes M Terroso G Figueiredo M . Depression and anxiety in systemic lupus erythematosus. Medicine. (2018) 97:e11376. doi: 10.1097/md.0000000000011376, PMID: 29995777 PMC6076116

[11] Yilmaz-Oner S Ilhan B Can M Alibaz-Oner F Polat-Korkmaz O Ozen G . Fatigue in systemic lupus erythematosus. Z Rheumatol. (2016) 76:913–9. doi: 10.1007/s00393-016-0185-0, PMID: 27600110

[12] Jump RL Robinson ME Armstrong AE Barnes EV Kilbourn KM Richards HB. Fatigue in systemic lupus erythematosus: contributions of disease activity, pain, depression, and perceived social support. J Rheumatol (2005) 32:1699–1705. PubMed Central PMCID: PMC16142863. 16142863

[13] Pinto B Dhooria A Grover S Jolly M Raj JM Sharma A. Fatigue and its correlates in Indian patients with systemic lupus erythematosus. Clin Rheumatol. (2020) 40:905–11. doi: 10.1007/s10067-020-05445-1, PMID: 33033858

[14] Fonseca R Bernardes M Terroso G de Sousa M Figueiredo-Braga M. Silent Burdens in Disease: Fatigue and Depression in SLE. Autoimmune Diseases. (2014) 2014:1–9. doi: 10.1155/2014/790724, PMID: 24592329 PMC3926392

[15] Nowicka-Sauer K Hajduk A Kujawska-Danecka H Banaszkiewicz D Smoleńska Ż Czuszyńska Z . Illness perception is significantly determined by depression and anxiety in systemic lupus erythematosus. Lupus. (2018) 27:454–60. doi: 10.1177/0961203317751858, PMID: 29325492

[16] Mertz P Piga M Chessa E Amoura Z Voll RE Schwarting A . Fatigue is independently associated with disease activity assessed using the Physician Global Assessment but not the SLEDAI in patients with systemic lupus erythematosus. RMD Open. (2022) 8:e002395. doi: 10.1136/rmdopen-2022-002395, PMID: 36123013 PMC9486369

[17] Du X Zhao Q Zhuang Y Chen H Shen B. Fatigue of systemic lupus erythematosus in China: contributors and effects on the quality of life. Patient Prefer Adherence. (2018) 12:1729–35. doi: 10.2147/ppa.S170984, PMID: 30233152 PMC6134956

[18] Arnaud L Gavand PE Voll R Schwarting A Maurier F Blaison G . Predictors of fatigue and severe fatigue in a large international cohort of patients with systemic lupus erythematosus and a systematic review of the literature. Rheumatology. (2019) 58:987–96. doi: 10.1093/rheumatology/key398, PMID: 30597077

[19] Feng C Zhao S Wu Y Li R Liu Y He X. Fatigue of systemic lupus erythematosus patients and its influencing factors. Chin Med Herald. (2019) 16:54–7.

[20] Omdal R Mellgren SI Koldingsnes W Jacobsen EA Husby G. Fatigue in patients with systemic lupus erythematosus: lack of associations to serum cytokines, antiphospholipid antibodies, or other disease characteristics. J Rheumatol. (2002) 29:482–6. 11908560

[21] Lu Y Jin X Feng L-W Tang C Neo M Ho RC. Effects of illness perception on negative emotions and fatigue in chronic rheumatic diseases: Rumination as a possible mediator. World J Clin Cases. (2022) 10:12515–31. doi: 10.12998/wjcc.v10.i34.12515, PMID: 36579115 PMC9791537

[22] Bower JE. Behavioral Symptoms in Patients With Breast Cancer and Survivors. J Clin Oncol. (2008) 26:768–77. doi: 10.1200/jco.2007.14.3248, PMID: 18258985 PMC3057774

[23] Karol DE Criscione-Schreiber LG Lin M Clowse MEB. Depressive symptoms and associated factors in systemic lupus erythematosus. Psychosomatics. (2013) 54:443–50. doi: 10.1016/j.psym.2012.09.004, PMID: 23274009

[24] Krupp LB LaRocca NG Muir-Nash J Steinberg AD. The fatigue severity scale. Application to patients with multiple sclerosis and systemic lupus erythematosus. Arch Neurol. (1989) 46:1121–3. doi: 10.1001/archneur.1989.00520460115022, PMID: 2803071

[25] Friedman JH Alves G Hagell P Marinus J Marsh L Martinez-Martin P . Fatigue rating scales critique and recommendations by the Movement Disorders Society task force on rating scales for Parkinson's disease. Mov Disord. (2010) 25:805–22. doi: 10.1002/mds.22989, PMID: 20461797

[26] Wu C Wang D. Clinical application and assessment of the Chinese version of Fatigue Severity Scale in stroke patients. Chin J Phys Med Rehabil. (2007) 29:608–11. doi: 10.3760/j.issn:0254-1424.2007.09.009

[27] Mei Y Li H Yang Y Su D Ma L Zhang T . Reliability and validity of Chinese version of the brief illness perception questionnaire in patients with breast cancer. J Nurs. (2015) 22:11–4. doi: 10.16460/j.issn1008-9969.2015.24.011

[28] Broadbent E Petrie KJ Main J Weinman J. The Brief Illness Perception Questionnaire. J Psychosom Res. (2006) 60:631–7. doi: 10.1016/j.jpsychores.2005.10.020, PMID: 16731240

[29] Liu X Tang M Hu L Wang A Wu H Zhao G . Reliability and validity of the Pittsburgh sleep quality index. Chin J Psychiatry. (1996) 29:103–7.

[30] Buysse DJ Reynolds CF 3rd Monk TH Berman SR Kupfer DJ. The Pittsburgh Sleep Quality Index: a new instrument for psychiatric practice and research. Psychiatry Res. (1989) 28:193–213. doi: 10.1016/0165-1781(89)90047-4, PMID: 2748771

[31] Wondie Y Mehnert A Hinz A. The hospital anxiety and depression scale (HADS) applied to Ethiopian cancer patients. PLoS One. (2020) 15:e0243357. doi: 10.1371/journal.pone.0243357, PMID: 33270779 PMC7714130

[32] Sun Z Liu H Jiao L Zhou T Yang L Fan J. Reliability and validity of hospital anxiety and depression scale. Chin J Clinicians (Electronic Edition). (2017) 11:198–201. doi: 10.3877/cma.j.issn.1674-0785.2017.02.005

[33] Qu N-n Li K-j. Study on the reliability and validity of international physical activity questionnaire (Chinese Vision,IPAQ). Chin J Epidemiol. (2004) 25:265–8. doi: 10.3760/j.issn:0254-6450.2004.03.021 15200945

[34] Fan M Lyu J He P. Chinese guidelines for data processing and analysis concerning the International Physical Activity Questionnaire. Chin J Epidemiol. (2014) 35:961–4. doi: 10.3760/cma.j.issn.0254-6450.2014.08.019 25376692

[35] Ciccozzi M Margiotta DPE Basta F Dolcini G Batani V Lo Vullo M . Physical activity and sedentary behavior in patients with systemic lupus erythematosus. PLoS One. (2018) 13:e0193728. doi: 10.1371/journal.pone.0193728 29505598 PMC5837187

[36] Macfarlane DJ Lee CCY Ho EYK Chan KL Chan DTS. Reliability and validity of the Chinese version of IPAQ (short, last 7 days). J Sci Med Sport. (2007) 10:45–51. doi: 10.1016/j.jsams.2006.05.003, PMID: 16807105

[37] Zimet GD Powell SS Farley GK Werkman S Berkoff KA. Psychometric characteristics of the Multidimensional Scale of Perceived Social Support. J Pers Assess. (1990) 55:610–7. doi: 10.1080/00223891.1990.9674095, PMID: 2280326

[38] Zhang F Zhu S Deng P. Evaluation of Perceived Social Support Scale used in study of social support among hospitalized patients in China. Chin Nurs Res. (2018) 32:2048–52. doi: 10.12102/j.issn.1009-6493.2018.13.015

[39] Moldovan I Cooray D Carr F Katsaros E Torralba K Shinada S . Pain and depression predict self-reported fatigue/energy in lupus. Lupus. (2013) 22:684–9. doi: 10.1177/0961203313486948, PMID: 23660302

[40] Lau CS Mak A. The socioeconomic burden of SLE. Nat Rev Rheumatol. (2009) 5:400–4. doi: 10.1038/nrrheum.2009.106, PMID: 19506585

[41] Ausserwinkler M Flamm M Gensluckner S Bogensberger K Paulweber B Trinka E . Exploring the link between socioeconomic factors and rheumatoid arthritis: Insights from a large Austrian study. Ann Epidemiol. (2025) 110:66–71. doi: 10.1016/j.annepidem.2025.07.025, PMID: 40706886

[42] li H Du Q Wang S Guan S Zhan H Tian W . The application and influence factors of FACIT fatigue scale in SLE patients. Natl Med J China. (2017) 97:2775–8. doi: 10.3760/cma.j.issn.0376-2491.2017.35.014, PMID: 28954338

[43] Tench CM. Fatigue in systemic lupus erythematosus: a randomized controlled trial of exercise. Rheumatology. (2003) 42:1050–4. doi: 10.1093/rheumatology/keg289, PMID: 12730519

[44] Robb-Nicholson LC Daltroy L Eaton H Gall V Wright E Hartley LH . Effects of aerobic conditioning in lupus fatigue: a pilot study. Br J Rheumatol. (1989) 28:500–5. doi: 10.1093/rheumatology/28.6.500 2590802

[45] Omdal R Sjöholm H Koldingsnes W Sundsfjord JA Jacobsen EA Husby G . Fatigue in patients with lupus is not associated with disturbances in cerebral blood flow as detected by SPECT. J Neurol. (2005) 252:78–83. doi: 10.1007/s00415-005-0610-9 15654558

[46] Costa DD Dritsa M Bernatsky S Pineau C Ménard HA Dasgupta K . Dimensions of fatigue in systemic lupus erythematosus: relationship to disease status and behavioral and psychosocial factors. J Rheumatol. (2006) 33:1282–8. 16758508

[47] Inoue M Shiozawa K Yoshihara R Yamane T Shima Y Hirano T . Predictors of poor sleep quality in patients with systemic lupus erythematosus. Clin Rheumatol. (2017) 36:1053–62. doi: 10.1007/s10067-017-3545-5, PMID: 28138857

[48] Mahieu MA Ahn GE Chmiel JS Dunlop DD Helenowski IB Semanik P . Fatigue, patient reported outcomes, and objective measurement of physical activity in systemic lupus erythematosus. Lupus. (2016) 25:1190–9. doi: 10.1177/0961203316631632, PMID: 26869353 PMC4980272

[49] Brown SE Shah A Czuber-Dochan W Bench S Stayt L. Non-pharmacological interventions for self-management of fatigue in adults: An umbrella review of potential interventions to support patients recovering from critical illness. J Crit Care. (2023) 75:75. doi: 10.1016/j.jcrc.2023.154279, PMID: 36828754

[50] Hagger MS Koch S Chatzisarantis NLD Orbell S. The common sense model of self-regulation: meta-analysis and test of a process model. Psychol Bull. (2017) 143:1117–54. doi: 10.1037/bul0000118, PMID: 28805401

[51] Primdahl J Hegelund A Lorenzen AG Loeppenthin K Dures E Appel Esbensen B. The experience of people with rheumatoid arthritis living with fatigue: a qualitative metasynthesis. BMJ Open. (2019) 9:e024338. doi: 10.1136/bmjopen-2018-024338, PMID: 30898808 PMC6475175

[52] Magrin ME D'Addario M Greco A Miglioretti M Sarini M Scrignaro M . Social support and adherence to treatment in hypertensive patients: a meta-analysis. Ann Behav Med. (2015) 49:307–18. doi: 10.1007/s12160-014-9663-2, PMID: 25341642

[53] Feng G Peng J Tu D Zheng JZ Feng C. Two Paradoxes in Linear Regression Analysis. Shanghai Arch Psychiatry. (2016) 28:355–60. doi: 10.11919/j.issn.1002-0829.216084, PMID: 28638214 PMC5434296

